# *Aspergillus fumigatus* Invasion Increases with Progressive Airway Ischemia

**DOI:** 10.1371/journal.pone.0077136

**Published:** 2013-10-14

**Authors:** Joe L. Hsu, Mohammad A. Khan, Raymond A. Sobel, Xinguo Jiang, Karl V. Clemons, Tom T. Nguyen, David A. Stevens, Marife Martinez, Mark R. Nicolls

**Affiliations:** 1 Division of Pulmonary and Critical Care Medicine, Department of Medicine, Stanford University School of Medicine, Stanford, California, United States of America; 2 Veterans Affairs Palo Alto Health Care System, Medical Service, Palo Alto, California, United States of America; 3 Veterans Affairs Palo Alto Health Care System, Pathology and Laboratory Service, Palo Alto, California, United States of America; 4 Department of Pathology, Stanford University School of Medicine, Stanford, California, United States of America; 5 Infectious Diseases Research Laboratory, California Institute for Medical Research, San Jose, California, United States of America; 6 Department of Medicine, Division of Infectious Diseases and Geographic Medicine, Stanford University School of Medicine, Stanford, California, United States of America; University of Wisconsin Medical School, United States of America

## Abstract

Despite the prevalence of *Aspergillus*-related disease in immune suppressed lung transplant patients, little is known of the host-pathogen interaction. Because of the mould’s angiotropic nature and because of its capacity to thrive in hypoxic conditions, we hypothesized that the degree of *Aspergillus* invasion would increase with progressive rejection-mediated ischemia of the allograft. To study this relationship, we utilized a novel orthotopic tracheal transplant model of *Aspergillus* infection, in which it was possible to assess the effects of tissue hypoxia and ischemia on airway infectivity. Laser Doppler flowmetry and FITC-lectin were used to determine blood perfusion, and a fiber optic microsensor was used to measure airway tissue oxygen tension. Fungal burden and depth of invasion were graded using histopathology. We demonstrated a high efficacy (80%) for producing a localized fungal tracheal infection with the majority of infection occurring at the donor-recipient anastomosis; *Aspergillus* was more invasive in allogeneic compared to syngeneic groups. During the study period, the overall kinetics of both non-infected and infected allografts was similar, demonstrating a progressive loss of perfusion and oxygenation, which reached a nadir by days 10-12 post-transplantation. The extent of *Aspergillus* invasion directly correlated with the degree of graft hypoxia and ischemia. Compared to the midtrachea, the donor-recipient anastomotic site exhibited lower perfusion and more invasive disease; a finding consistent with clinical experience. For the first time, we identify ischemia as a putative risk factor for *Aspergillus* invasion. Therapeutic approaches focused on preserving vascular health may play an important role in limiting *Aspergillus* infections.

## Introduction

*Aspergillus fumigatus* is a ubiquitous mould that grows in decaying organic matter and releases airborne spores, which can result in a variety of respiratory tract infections including persistent colonization, allergic bronchopulmonary aspergillosis (ABPA), *Aspergillus* tracheobronchitis, chronic necrotizing *Aspergillus pneumonia* and invasive pulmonary aspergillosis (IPA) [1]. The presentation of these lung diseases varies according to the comorbid status of the host [1]. For example, patients with cystic fibrosis and asthma often are affected by *Aspergillus* colonization or ABPA but rarely develop IPA [1,2]. *Aspergillus* colonization is common in chronic obstructive pulmonary disease (COPD), and less commonly chronic necrotizing pneumonia and IPA are also seen [1,3]. For lung transplant recipients, infection with *A. fumigatus* represents a major cause of morbidity, with mortality rates as high as 82% [4-7]. In addition to IPA, lung transplant patients are at risk for *A. fumigatus* infections of the tracheal anastomosis, resulting in serious bronchial complications and chronic graft rejection, which has been associated with *Aspergillus* colonization [5,8,9]. Despite the considerable morbidity of these infections, little is known of the host-pathogen interactions that predispose persons to the wide range of *Aspergillus*-related pulmonary diseases. 

*Aspergillus*, while considered a facultative aerobe, can grow in severely hypoxic conditions at oxygen levels as low as 0.1% (0.76 mmHg) [10]. Recently, researchers have begun to elucidate the capacity of *Aspergillus* to adapt to hypoxic environments that may allow the pathogen to modulate the host inflammatory response [11]. Additionally, studies have implicated gliotoxin, a secondary metabolite of *Aspergillus*, as capable of inhibiting host angiogenesis [12]. Together, these observations suggest a dynamic host-pathogen interaction whereby the fungus adapts to and simultaneously induces a favorable ischemic microenvironment; a property that likely confers an advantage in virulence. 

Lung transplants are particularly vulnerable to the effects of graft ischemia, as they are the only solid organ allograft that does not undergo primary systemic arterial revascularization (i.e., bronchial artery restoration) at the time of surgery. This results in a prolonged period of relative hypoxia in the grafted organ [13]. Following surgery, transplant airways are presumed to rely principally on the pulmonary artery microcirculation and these vessels display foreign HLA antigens making them a target for allospecific immunity. In rejection, microvascular injury leads to tissue infarction [14]; these ischemic zones may provide a substrate for the growth of microorganisms analogous to the devascularized tissues of diabetic patients, promoting chronic infections. Yet, there is a paucity of information regarding the risk factors that predispose lung transplant patients to fungal infections, the effect of vascular ischemia on the pathogenesis of *Aspergillus* infections, or the role that the bronchial circulation may play in maintaining the host’s defense mechanism [15]. Because of this mould’s angiotropic nature and capacity to thrive under hypoxic conditions [10-12,16], we hypothesized that the degree of *Aspergillus* invasion would increase with the progressive ischemia of the rejecting allograft. 

We have developed a technique for airway transplantation, known as orthotopic tracheal transplantation (OTT). The OTT model, due to the planar anatomy of the trachea, is an ideal model for studying the microvasculature in transplantation because of how blood vessels are linearly displayed in two dimensions, in contrast to the complex anatomy of vessels in terminal airways, which are difficult to capture by histological techniques. Our research group has previously demonstrated that acutely rejecting tracheal transplants undergo a series of distinct microvascular events including: 1) reperfusion of the donor graft from reanastomosis with recipient vessels that is partially dependent on hypoxia inducible factor (HIF) pathways [17], 2) profound ischemia due to CD4^+^ T cells and complement-mediated rejection [18], and 3) recipient-derived neovascularization [17]. 

In the current study, we utilized the OTT model of *Aspergillus* infection to evaluate the role that microvascular ischemia plays in promoting *Aspergillus* invasion. We show that the *Aspergillus*-OTT model mimics the tracheal infections observed clinically in lung transplant patients. We observe for the first time, that the depth of *A. fumigatus* invasion correlated with progressive rejection-mediated vascular ischemia and hypoxia. *Aspergillus* infection also correlated with a decrease in regional blood perfusion. By elucidating the complex host-pathogen interaction that may contribute to this clinical dilemma for lung transplant recipients, this model has the potential to facilitate the development of novel therapeutic concepts that may help prevent or improve the treatment of fungal infections in lung transplant recipients. 

## Methods

### Experimental design

All animal studies were performed with the approval of the Veterans Affairs Palo Alto Heath Care System’s Institutional Animal Care and Use Committee (protocol number NIM1483) under the guidelines for the care and use of laboratory animals from the Office of Laboratory Animal Welfare of the National Institutes for Health. In addition, The Stanford University Applied Panel on Biosafety (protocol number 1007-MN0312) approved all microbiological experiments performed in this study. All research was conducted according to the ethical and regulatory standards as outlined in the *U.S. Government Principles for the Utilization and Care of Vertebrate Animals Used in Testing*, *Research and Training* (prepared by the Interagency Research Animal Committee), the *Guide for the Care and Use of Laboratory Animals* (prepared by the National Research Council) and the *AVMA Guidelines on Euthanasia*. Five-week old male C57BL/6 and BALB/c mice were purchased from Jackson Laboratories. Groups consisted of >4 mice in all experiments. We initially developed the OTT *A. fumigatus* infection model in allogeneic transplants (BALB/c donor to B6 recipient) compared with syngeneic transplants (B6 donor and recipient). Through an iterative process, we determined inoculation route and conidial quantity sufficient to produce a localized tracheal infection in >80% of animals. 

We initially infected syngeneic and allogeneic animals on day 10 post-transplant. Subsequent studies evaluated allogeneic animals infected on days 3, 6, and 10 post-transplant. For perfusion and tissue oxygen tension experiments, animals were evaluated on post-infection days 1 and 2 (n = 4-8 animals/time point). For example, an animal infected on post-transplant day 3 was evaluated on post-infection days 1 and 2, representing the animal’s post-transplant day 4 and 5, respectively. Non-infected animals were evaluated at 2-day intervals post-transplantation (n = 4-8 animals/time point). For FITC-lectin studies infected animals were evaluated 2 days post-infection and compared to non-infected animals on the equivalent post-transplant day (n = 3-5 animals/group). For example, an animal infected on day 3 post-transplant was studied with FITC-lectin on day 5 post-transplant (post-infection day 2) and compared to a non-infected control on post-transplant day 5. Initially, we compared regional perfusion differences in infected and non-infected animals post-transplant (syngeneic and allogeneic) day 8 (n = 4-8 animals/experimental group). We then evaluated regional perfusion differences in infected allografts on post-transplant days 5 and 12. Fungal burden and depth of fungal invasion were measured histopathologically on post-infection day 2 (n = 7-11 animals/group). These animals were compared to non-transplanted controls treated with triamcinolone acetonide and inoculated with *A. fumigatus* (n = 6 animals/group). For additional details of the experimental approach see Figure S1.

### Orthotopic tracheal transplant

**Figure 1:**
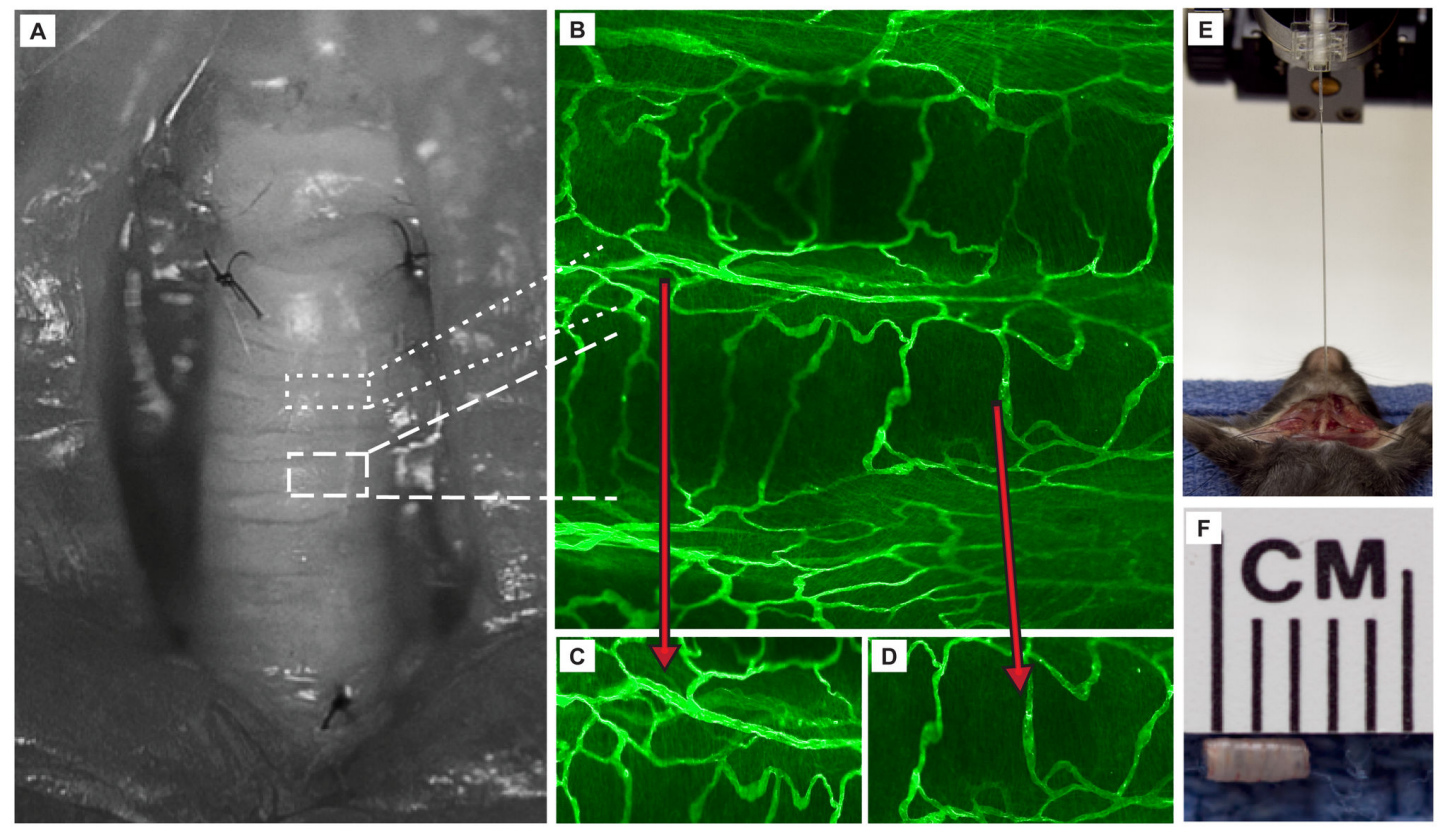
Orthotopic tracheal transplant: method of assessment for microvascular perfusion and tissue hypoxia. (**A**) The OTT involves the removal of a 7-ring donor segment (shown in panel F) and transplantation into an allogeneic animal (BALB/cB6) or syngeneic animal (B6B6) under the microscope. Two sutures (10-0 nylon) are placed in the cephalad anastomosis and 3 sutures secure the caudad anastomosis. (**B**) FITC-lectin perfusion profile of whole-mount tracheal grafts in un-transplanted control illustrates the linear display of blood vessels in 2-dimensions. (**C**) OTT vessels in the intra-membraneous tracheal portion. (**D**) OTT vessels in the cartilaginous portion of the tracheal segment. (**E**) Illustrative example of the method for measurement of perfusion, using a laser Doppler flowmetry probe, and tissue oxygen tension measurement using a microsensor tissue oxygenation probe. (**F**) Gross image of explanted tracheal segment, dimensions are approximately width, 1mm x length, 2.5 mm.

Transplants were performed, as previously described [17,18]. Briefly, a seven-ring tracheal segment was removed from a CO_2_-euthanized donor. Recipient mice were anesthetized with 50 mg/kg ketamine and 10 mg/kg xylazine, and a short incision was made at the midline neck region. After the recipient’s trachea was transected, the donor trachea was sewn in with 10-0 nylon sutures (2 sutures at the cephalad and 3 sutures at the caudal anastomoses, respectively) and the overlying skin was closed with 5-0 silk (Figure 1A). 

### *Aspergillus fumigatus* infection model

*Aspergillus fumigatus*, 10AF (ATCC 90240) was used as the challenge organism for all infections. Cultures were revived from long-term storage at -80°C by growth at 37°C on potato dextrose agar. Conidia were harvested by washing the surface of the agar plate with 0.05% Tween 80 in saline (v/v). The conidial suspension was vortexed to disperse clumps of conidia and stored at 4°C until needed. Viability was assessed by quantitative plating of serial dilutions of the conidia onto Sabouraud dextrose agar (SDA) plates. These were incubated for 48 hours at 37°C and the number of colonies counted. The suspension was then diluted to the desired number of conidia per ml. Prior to infection conidia were centrifuged (5000 x g for 5 min) and then washed twice with 1X PBS after centrifugation to remove excess Tween. Transplanted mice were inoculated with *A. fumigatus* (3-4 x 10^8^ conidia/ml in 40 µl volume) while under anesthesia (ketamine/xylazine). Inoculation occurred via *direct tracheal inoculation* in which 40 μl of the conidial solution was placed inside the trachea and allowed to dwell for 1 hour, prior to transplantation. Inoculation also occurred by *intra-tracheal injection*, in which a 40 μl conidial solution was injected at a point 2 tracheal rings rostral to the cephalad anastomosis. For this procedure we used a 29-gauge insulin syringe. After inoculation, all animals, including controls, were administered triamcinolone acetonide (40 mg/kg) subcutaneously. Clinical observations were noted and weights determined daily. At day 2 after infection, all surviving mice were euthanatized using CO_2_ asphyxia. 

### Measurement of microvascular perfusion and luminal oxygen tension

Prior to euthanasia, we determined the degree of microvascular perfusion and tissue oxygen tension of the graft, as described previously [17-19]. Briefly, OTT recipients were anesthetized (ketamine/xylazine), the trachea was exposed and a Laser Doppler flowmetry probe was placed abutting the anterior membranous portion between tracheal rings, using a micromanipulator (Figure 1E). For each location of interest nine serial, blood perfusion unit (BPU) measurements (right, center, and left, in triplicate) were obtained. For tissue oxygen tension (pO_2_) measurements a small hole was made in the ventral trachea within 2 rings of the anastomosis. Serial measurements were taken as described elsewhere [18,19]. For infected allografts, blood perfusion and tissue pO_2_ were measured at 24 hours and 48 hours after infection. Thus, measurements for infected allografts were obtained on days 4, 5, 7, 8, 11 and 12 post-transplantation. For non-infected controls, we obtained measurements at two-day intervals. 

### FITC-lectin microvascular perfusion studies

In addition, we performed FITC-lectin perfusion studies as previously described [17-19]. This procedure was used to confirm whether vessels were functional and continuous with the systemic circulation. Briefly, 100 μl of 1mg/mL lectin (Vector Laboratories, Burlingame, CA) was injected by direct cardiac puncture with a 29-gauge needle over one minute. After three minutes of circulation, a sternotomy was performed followed by right atriotomy and cannulation of the aorta via the left ventricle with an 18-gauge angiocatheter. One percent paraformaldehyde in PBS was perfused via the aorta for 2 minutes at 120 mmHg. The orthotopic tracheal graft along with adjoining recipient trachea was dissected from the surrounding tissues. The trachea was removed with both recipient ends and divided along the ventral midline, creating a flat piece of tissue, and was processed for confocal microscopy (Figure 1B-D). 

### Histological preparation and evaluation of tracheal samples

The presence and extent of fungal invasion was determined by histology. All tracheal samples were placed in paraffin blocks and cut longitudinally or transversely in 5-μm sections through the entire tracheal segment. Grocott’s methenamine silver (GMS) staining was performed for each of these sections (Histotech laboratories, Hayward, CA). For measurements of both fungal burden and invasion the most severe section was scored for each tracheal sample. On average per tracheal sample the number of fields evaluated histologically with a GMS-stain were 180 for longitudinal sections and 400 fields for transverse sections. An experienced clinician (JH) evaluated all histological slides microscopically. A board certified anatomic pathologist (RS), blinded to experimental group, graded fungal burden and invasion. Differences in grading were adjudicated by a consensus score decided jointly by reviewers (JH and RS). Fungal infection was defined by the presence of hyphal elements on GMS stain in the tracheal segment. The degree of fungal invasion was semiquantitatively graded: 1 (minimal) invasion of the epithelial layer, 2 (mild) invasion of the subepithelial layer, 3 (moderate), invasion to the depth of the cartilaginous tracheal ring, and 4 (severe), invasion through the tracheal wall with abluminal evidence of fungal infection (Figure S2A). The degree of fungal burden was determined using a semiquantitative scoring system as follows: 0, no fungal elements, 1 (minimal), fungal hyphae in less than 25% of the luminal area, 2 (mild) hyphae occluding 25% to 49% of the tracheal area, 3 (moderate) hyphae 50% to 74% of tracheal area, and 4 (severe) greater than 75% occlusion of the tracheal area (Figure S2B). 

### Determination of disseminated infection

To determine dissemination of infection we quantified fungal burden in the kidney and lung using colony forming units (CFU) and quantitative polymerase chain reaction (qPCR). The fungal burden in organs was determined by quantitative plating of organ homogenates as described previously [20]. Organs were weighed and placed in 5 ml saline with penicillin and streptomycin in a Whirlpak bag and homogenized. Additional serial dilutions were made and samples plated onto SDA with 50 mg of chloramphenicol per liter. In addition, the remaining sample of each homogenate was frozen at -80°C for DNA extraction for qPCR determination of the burden of *A. fumigatus*. After mechanical breakage of the homogenate using 0.5 mm zirconia beads in a Beadbeater (BioSpec, Bartlesville, OK), DNA was extracted using a QiAmp DNA Mini Kit (Qiagen Inc, Valencia, CA) per manufacturer’s instructions. Quantitative PCR was performed in triplicate samples using the following primers of the 28S rRNA of *A. fumigatus*: 28S-466 (5’-CTC GGA ATG TAT CAC CTC TCG G-3’) and 28S-533 (5’-TCC TCG GTC CAG GCA GG-3’); and the Taqman probe was 28S-490 (5’-6-carboxyfluorescein-TGT CTT ATA GCC GAG GGT GCA ATG CG-3’-6-carboxy tetramethylrhodamine) [21]. DNA was amplified and fluorescence detected with a Thermocycler/ABI Prism 7700 sequence detector (Applied Biosystems, Carlsbad, CA) with the following cycle conditions: 95°C for 10 min followed by 45 amplification cycles (15 seconds of denaturation at 95°C and 1 min of hybridization and elongation at 60°C) [21]. Known concentrations of conidia/ml were spiked into naïve (uninfected) tissues (kidney and lung) and the equivalent cycle threshold (Ct) was used to create a standard conidial equivalent curve for the respective organ. Colony forming units and qPCR conidial equivalents were standardized by weight of the organ in grams. 

### Statistics

GraphPad Prism version 5.0c and SPSS were used for statistical analysis. Differences in BPU and tissue pO_2_ tension between infected and non-infected allografts were evaluated using an unpaired Student’s t-test. A paired t-test was used to compare the mean BPU and tissue oxygen tension differences between the cephalad anastomosis and the midtracheal segments in each animal. Inter-rater reliability of the semiquantitative scoring method for fungal burden and invasion were evaluated by a Kappa statistic, using SPSS. Degree of agreement was based on criteria developed by Fleiss [22]. Semiquantitative differences in fungal burden and depth of invasion by day post-transplant were evaluated by a non-parametric Kruskal-Wallis test. A Dunn’s multiple comparisons test was used to evaluate differences between days. All t-tests were two-tailed, and significance was judged at a level of p <0.05.

## Results

### High efficacy of tracheal infection in the OTT *A. fumigatus* model

**Figure 2:**
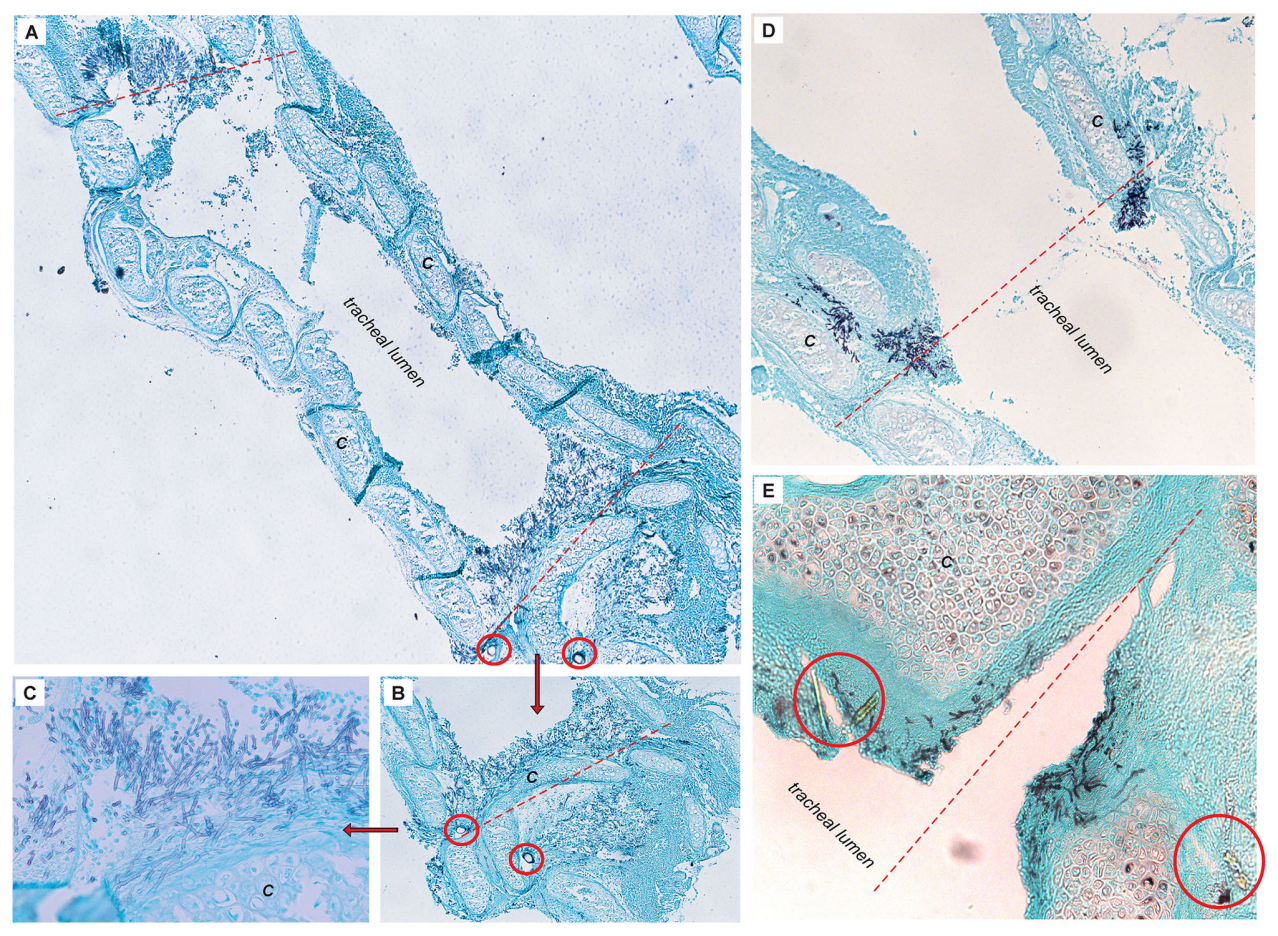
Predilection for anastomotic infection is independent of route of infection or transplant type. (**A**) Grocott’s methenamine silver stained histopathology of longitudinal section for a syngeneic (B6B6) animal infected with *A. fumigatus* by direct tracheal inoculation. Infection localized to both anastomoses (red dotted lines). Suture material (red circles) can be seen at inferior anastomosis (original magnification 5X). “*C*” denotes tracheal cartilaginous ring. (**B**) Syngeneic transplant inferior anastomosis, demonstrating aggressive abluminal invasion of fungus (20X magnification). (**C**) Syngeneic transplant inferior anastomotic infection (40X magnification). (**D**) Allogeneic animal (BALB/cB6) infected by intra-tracheal injection, demonstrating an aggressive infection at the anastomosis (red dotted line) (10X magnification). (**E**) Dehiscence of donor-recipient anastomosis in allogeneic animal infected via direct tracheal infection route with invasive hyphal elements (40x magnification). Suture material (red circles).

As an initial “proof of principle” study to establish a localized infection in the tracheal graft, we directly inoculated the tracheal segment and transplanted the graft into recipient allogeneic or syngeneic animals. On average 180 longitudinal sections or 400 transverse sections per tracheal sample were evaluated histologically with a GMS-stain. Using the technique of direct tracheal inoculation, we established an invasive infection of the tracheal lumen in all animals (Figure 2A-C,E). We then infected syngeneic and allogeneic transplanted animals by intra-tracheal injection. Using a 10^8^ conidia/ml inoculum, we consistently produced a histopathologically evident tracheal infection in 80% (8/10) of allogeneic transplants. By comparison, a 10^7^ conidial/ml inoculum produced an infection in 60% (3/5) of allogeneic animals. Once we demonstrated the efficacy of the intra-tracheal injection method all subsequent experiments utilized this form of inoculation. In these initial experiments, regardless of the route of infection (direct tracheal infection at the time of transplantation or intra-tracheal injection following transplantation) or transplant type (syngeneic or allogeneic), infection occurred primarily at the anastomosis site dividing the donor graft from the recipient (Figure 2). Moreover, the infection was largely confined to the grafted segment. Thus, this OTT model mimics the unique predilection for anastomotic *Aspergillus* infections observed clinically among lung transplant recipients [8]. 

### Allogeneic transplants demonstrate a more invasive *Aspergillus infection* than syngeneic transplants at day 12 post-transplantation

**Figure 3:**
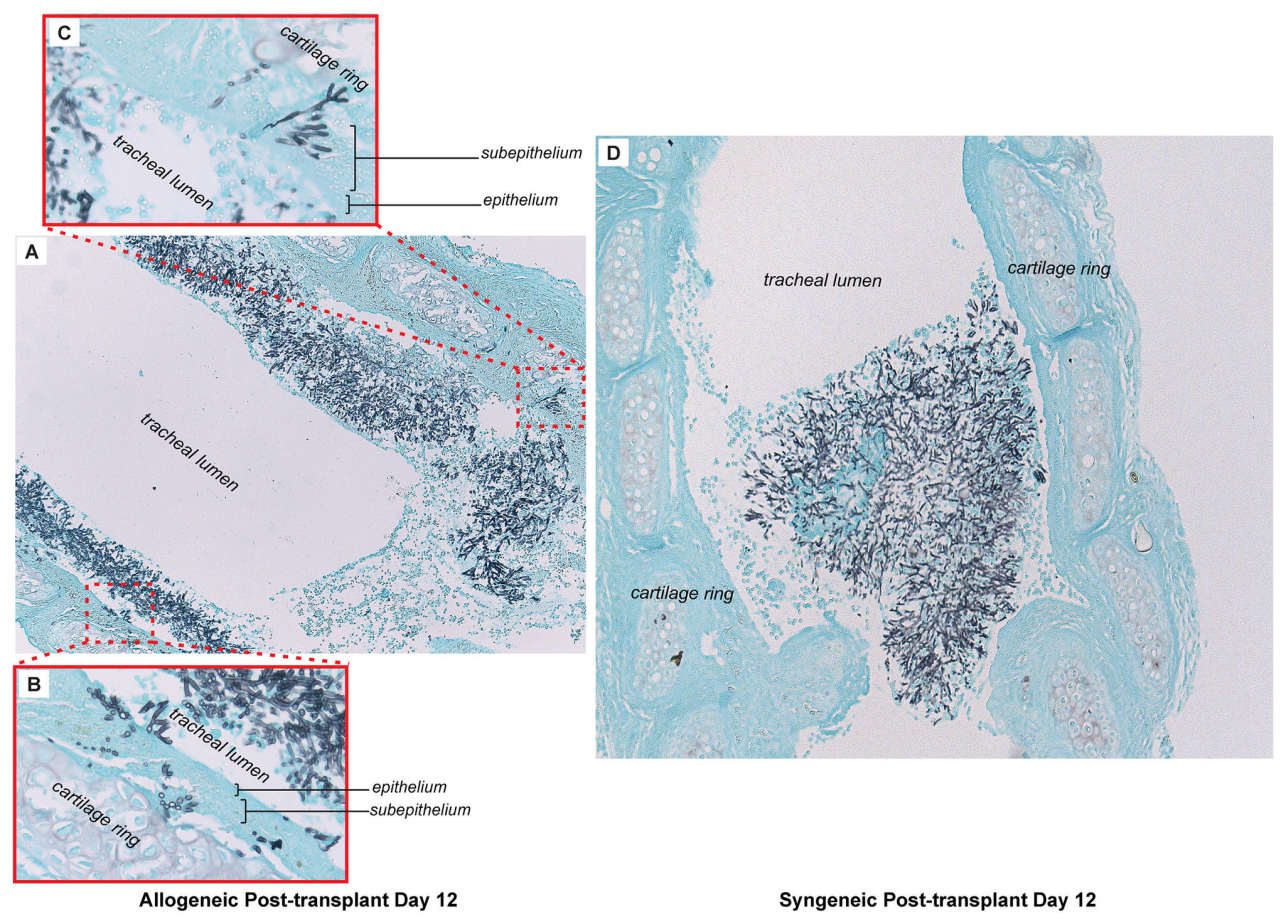
Allogeneic transplants demonstrate a more invasive *Aspergillus* infection compared to syngeneic transplants. (**A**) Grocott’s methenamine silver staining of longitudinal section in allogeneic animal at day 12 post-transplantation, demonstrating infection throughout tracheal segment (10X magnification). (**B**, **C**) Invasive fungal infection is to the level of the cartilaginous ring (40X magnification). (**D**) Longitudinal section of syngraft at day 12 post-transplantation shows high fungal burden occluding tracheal lumen without evidence of invasive infection (10X magnification).

We previously demonstrated that by day 12, allografts exhibited severe immune-mediated microvascular angiopathy, whereas syngrafts maintain their microvascular integrity [18]. To explore the hypothesis that *A. fumigatus* may be influenced by the graft’s microvascular health, we first sought to determine histological differences in *Aspergillus* infection between allogeneic and syngeneic transplants at day 12. Regardless of transplant type, hyphae were evident primarily at the donor-recipient anastomosis in 73% (8/11) of infected animals. For the remaining 3 animals, infection was present throughout the length of the graft. In the syngeneic transplant model, histopathology showed hyphal masses that completely occluded the tracheal lumen (Figure 3D). However, despite the high fungal burden, hyphal elements did not invade, or only minimally invaded, the luminal epithelium of syngeneic transplants. By contrast, the histopathology of the allogeneic transplants was characterized by an extensive fungal burden, and all samples displayed an aggressive hyphal invasion to the level of the tracheal cartilaginous ring, extending beyond the subepithelium (Figure 3A-C). Thus, allogeneic transplants experienced invasive *A. fumigatus* infections, whereas the infection in syngeneic transplants was analogous to *Aspergillus* airway colonization. 

### Infected and non-infected allografts display similar blood perfusion and overall tissue oxygen kinetics

**Figure 4:**
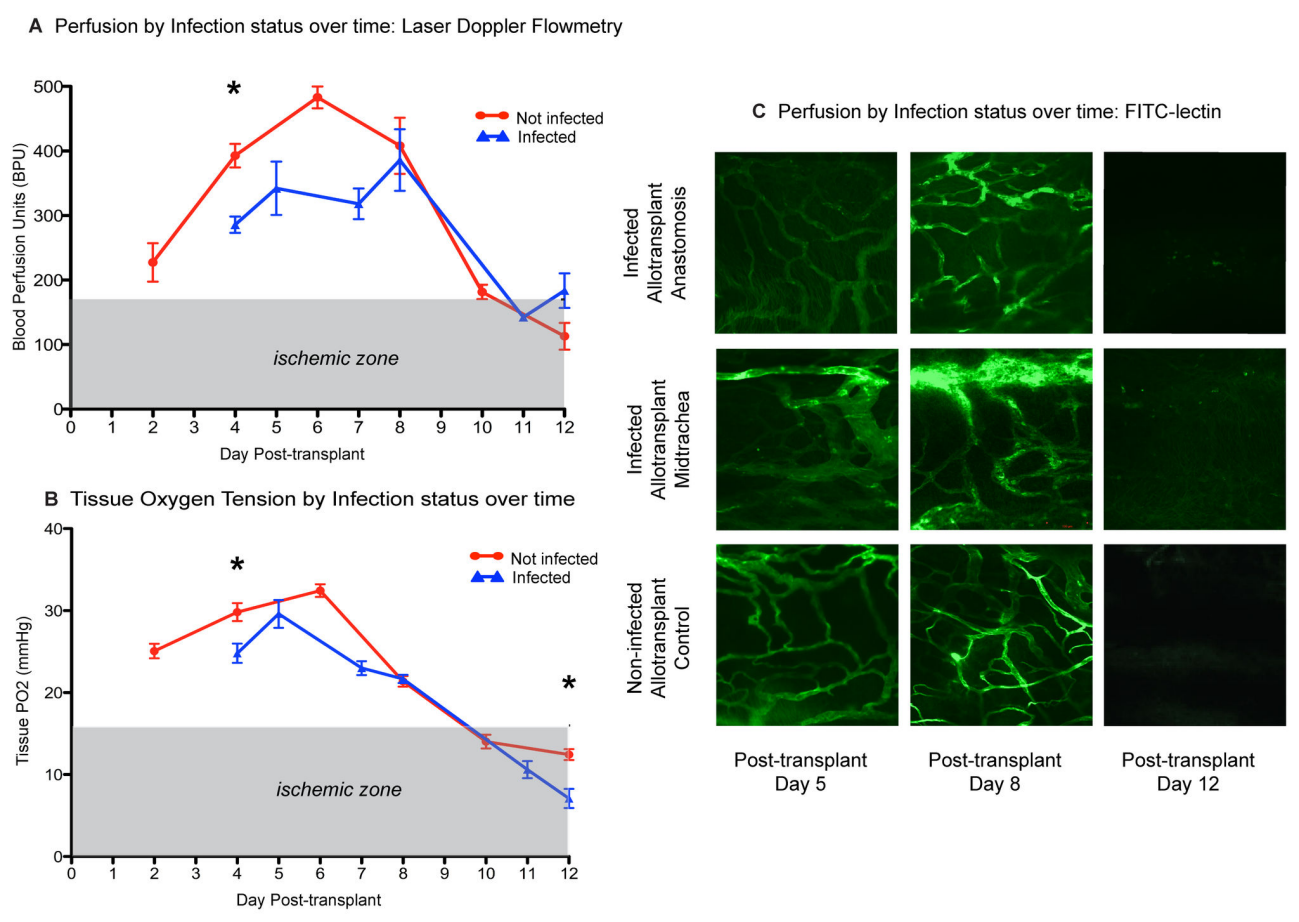
Infected and non-infected allografts share similar overall blood perfusion and luminal tissue oxygen kinetics. All measurements represent pooled data from animals on post-infection days 1 and 2. (**A**) Blood perfusion measurements (mean +/- SEM, BPU) plotted over time (n = 4-8 animals/time point) in infected (blue) and non-infected (red) allografts. Both show similar overall blood perfusion kinetics with an initial increase in graft perfusion followed by progressive microvascular ischemia between day 8 and day 12. “Ischemic zone” is based on previous studies [18] denoted by shaded area. (**B**) Tissue pO_2_ measurements (mean +/- SEM, mmHg) plotted over time (n = 4-7 animals/time point) in infected (blue) and non-infected (red) allografts, demonstrating similar overall tissue pO_2_ kinetics regardless of infection status. “Ischemic zone” is based on previous studies [18] denoted by shaded area. *p <0.05. (**C**) FITC-lectin perfusion profile of whole-mount tracheal grafts on days 5, 8, and 12 post-transplantation by infection status. Lower row represents non-infected allograft perfusion at donor-recipient anastomosis. For animals at day 5 and 8, the middle row depict microvasculature of the midtrachea in the infected allograft, demonstrating dilated vasculature compared with the vessels at the anastomosis (top row). Day 12 animals experience profound loss of microvasculature.

Because *A. fumigatus* has been shown to modulate host angiogenesis [12], we sought to characterize the effect that *Aspergillus* may have on blood perfusion and tissue pO_2_ kinetics in infected and non-infected allogeneic grafts at various days post-transplant. As previously observed by our group, we demonstrated that substantive changes in blood perfusion occur in the allograft at the following time points: days 3-5, an increased perfusion due to the reanastomosis of recipient and donor vessels; days 6-8, the onset of immune-mediated vascular rejection; and days 10-12, the time of maximal graft ischemia (Figure 4) [18]. Accordingly, to study the impact of these microvascular changes on *A. fumigatus* growth patterns, we chose to infect animals on days 3, 6 and 10 post-transplant. FITC-lectin studies were performed in infected and non-infected animals on days 5, 8 and 12 post-transplant. 

Blood perfusion differences between infected and non-infected allografts were measured using a Doppler flowmetry probe [18]. Overall kinetics of both infected and non-infected allografts were similar, demonstrating progressive microvascular rarefaction from day 8 to day 12 post-transplantation (Figure 4A). Blood perfusion units in these animals declined from 385.2 bpu to 183.8 bpu, and 408 bpu to 112.9 bpu in infected and non-infected allografts, respectively. In our previous work, BPU levels of 170 bpu or less were considered to be severely ischemic (Figure 4A, shaded area) [18]. A significantly lower blood perfusion for infected compared to non-infected allografts was observed only at day 4 (285.7 bpu and 392.8 bpu, respectively, p=0.003). The results from the Doppler flowmetry probe were consistent with the FITC-lectin perfusion studies performed on days 5, 8 and 12 (Figure 4C). In these studies, we demonstrate perfusion on day 5 and 8, which disappears by day 12. We observed a relative microvascular dilation of the midtracheal vessels compared to those at the anastomosis (Figure 4C). 

Confirming results from our previous studies, the luminal surface of the rejecting allograft undergoes progressive hypoxia starting at day 6 after transplantation [18]. Based on our previous research, oxygen levels below 16 mmHg (2.1%) were defined as severely ischemic (Figure 4B, shaded area) [18]. In non-infected allografts, oxygen tension decreased from day 6 (32 mmHg) to day 12 (12.4 mmHg) and infected allografts had levels of 23 mmHg and 7.1 mmHg on day 7 and 12, respectively (Figure 4B). Luminal oxygen tension was significantly lower in infected compared to non-infected allografts at day 4 (24.8 mmHg and 29.8 mmHg, respectively p=0.02) and day 12 (7.1 mmHg and 12.4 mmHg, respectively p=0.008). 

### Regional perfusion differences occur in *Aspergillus*-infected allografts

**Figure 5:**
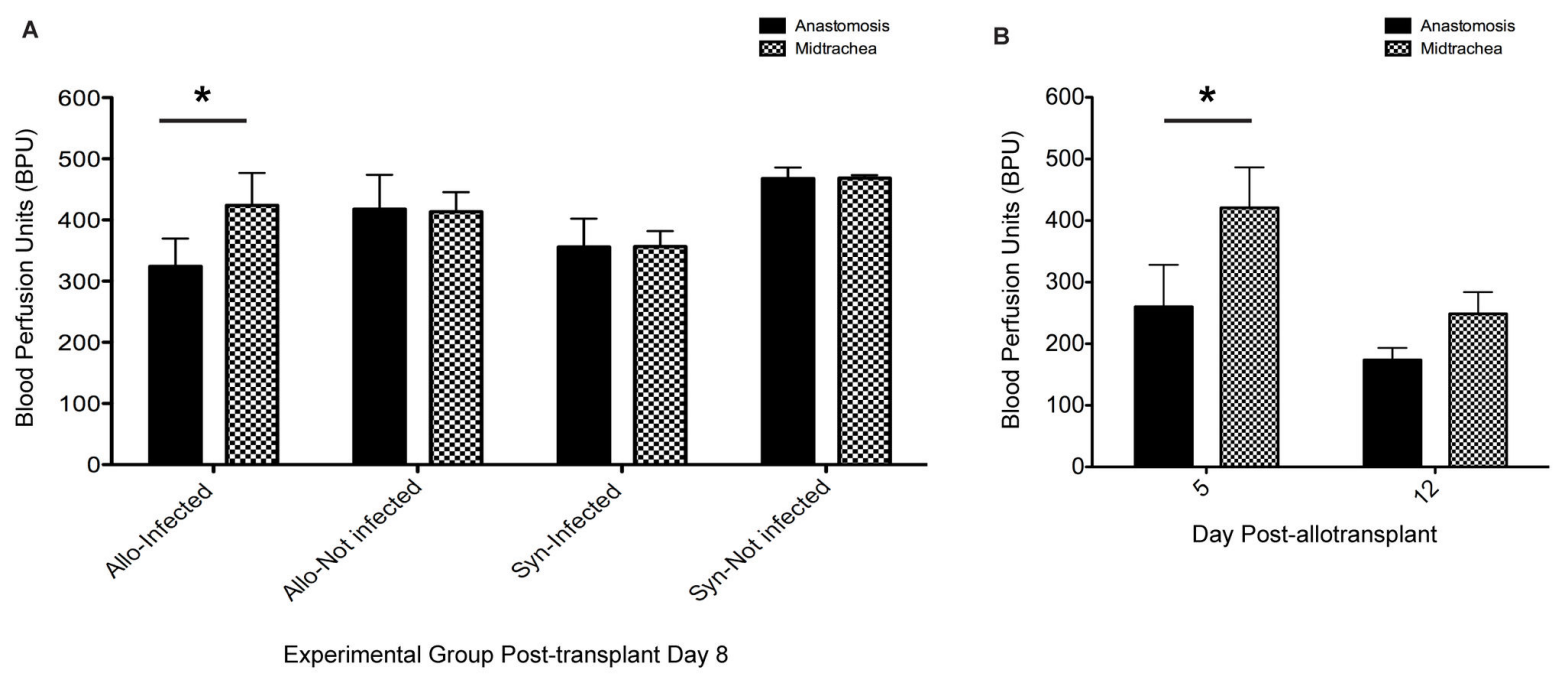
Decreased regional allograft perfusion at rejecting anastomosis correlates with location of *Aspergillus* infection. (**A**) Day 8 regional perfusion differences as measured by laser Doppler flowmetry (means, SEM, BPU), by transplant type and infection status. Only allografts demonstrate lower perfusion at the infected anastomosis compared with the midtrachea. (**B**) Laser Doppler flowmetry (means, SEM, BPU) shows regional perfusion differences for allografts at day 5 and 12 post-transplantation (n = 4-8 animals/experimental group). * p <0.05.

Because of the anastomotic predilection for infection observed in our initial studies (Figure 2), we evaluated regional blood perfusion differences between the cephalad anastomosis and the midtracheal segment by transplantation type and infection status for day 8 animals (Figure 5A). In infected animals regional perfusion differences were assessed on the second day of infection after inoculation on day 6 post-transplant. Our results demonstrate a significantly higher Doppler flowmetry blood perfusion in the midtracheal segment (424 bpu) compared with the anastomotic segment (324.3 bpu) for allograft animals at day 8 (p=0.03). Regional differences in BPU were not observed for non-infected animals or syngeneic transplants infected with *A. fumigatus*. To further evaluate the BPU differences in infected allografts, we measured regional perfusion differences by day post-transplantation (Figure 5B). The BPU for allografts at day 5 was 420.5 bpu and 259.9 bpu, for midtracheal and anastomotic segments, respectively (p=0.03). There were no significant regional perfusion differences observed in day 12 allotransplants.

### *Aspergillus fumigatus* invasion increases with allograft tissue ischemia

**Figure 6:**
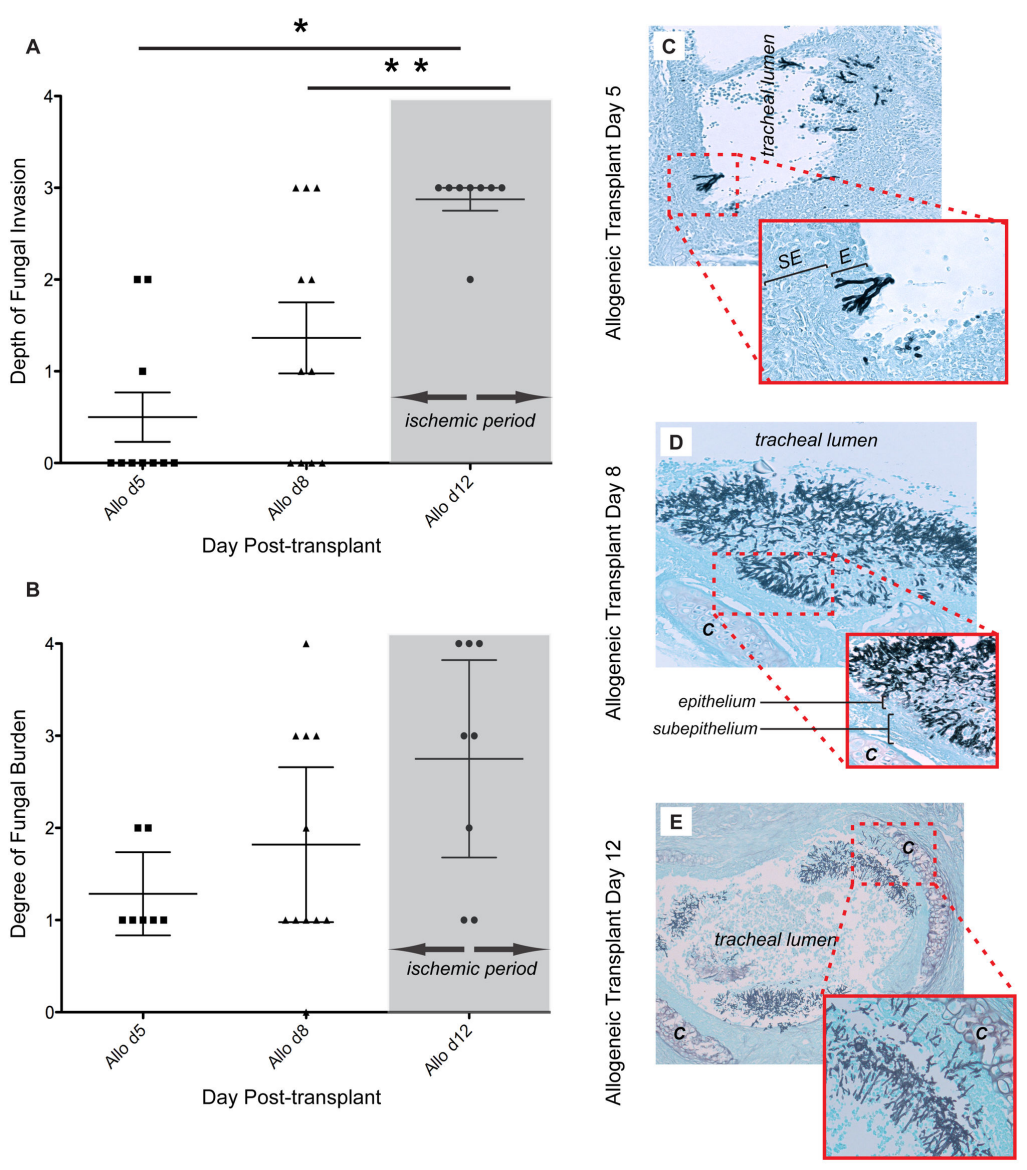
*Aspergillus fumigatus* invasion increases with allograft tissue ischemia. (**A**) Depth of fungal invasion, as measured on a semiquantitative scale 0-4 (Figure S2A, mean, 95% confidence interval (CI)), (n = 8-11 animals/time period) by day post-allotransplant. Mean depth of invasion increases from day 5 to day 12, as animal enters ischemic period (day 10-12, as illustrated by shaded gray area) [18]. *p<0.05 between days 5 and 12, **p<0.05 between days 8 and 12. (**B**) Degree of fungal burden, as measured on a semiquantitative scale 0-4 (Figure S2B, mean, 95% CI), (n = 7-11 animals/time period) by day post-allotransplant. Fungal burden increases, albeit not significantly, as animal enters ischemic period (day 10-12, as illustrated by shaded gray area) [18]. (**C**-**D**) Representative GMS-histopathology of longitudinal sections for (**C**) day 5 (10X magnification), inset (40X magnification) and (**D**) day 8 (20X magnification), inset (40X magnification). (**E**) Day 12 transverse section illustrates deeply invasive infection (grade 3) to the level of the cartilaginous ring (10x magnification) inset (20X magnification). “*C*” denotes cartilaginous ring, “*E*” specifies epithelial layer, “*SE*” specifies subepithelial layer.

To determine if the depth and burden of *Aspergillus* infection increase with rejection- mediated airway ischemia and hypoxia, we evaluated the histopathology of allografts at days 5, 8 and 12 (Figure 6) post-transplant. After 2 days of infection, animals at each time point were compared using a semiquantitative 4-point scale for fungal burden and depth of invasion (for histological examples of our semiquantitative scoring system see Figure S2). The inter-interpreter agreement for the scoring of histopathology slides, as measured by the Kappa statistic, was 0.82 for fungal burden and 0.71 for depth of invasion, suggesting an excellent level of inter-interpreter agreement [22]. Day 12 animals consistently demonstrated the highest grade of invasion observed (88% (7/8) grade 3). By contrast, no animals at day 5 (0/10), and 27% (3/11) at day 8 demonstrated a grade 3 depth of *A. fumigatus* invasion (Figure 6) (Kruskal-Wallis test, p=0.0006). Grade 4 invasion was not observed among these animals. Using qPCR and CFU of lung and kidney samples, we were not able to demonstrate an association between increased local invasion and systemic dissemination of *A. fumigatus* (Figure S3). Degree of fungal burden was highest in allografts at day 12 with 63% (5/8) with grade ≥ 3 compared to day 5 (0/10) or 8 (37% (4/11)) (Kruskal-Wallis test, p=0.07). Among infected animals *Aspergillus* hyphae were located primarily at the donor-recipient anastomosis on day 5: 89% (8/9), day 8: 90% (9/10) and day 12: 63% (5/8). For the remaining day 12 animals infection occurred throughout the grafted segment. In non-transplanted controls, injecting a 10^8^ conidial/ml suspension intra-tracheally, produced no luminal infections in the six animals tested. In summary, the capacity for *A. fumigatus* to cause invasive disease increased significantly following the microvascular destruction and ischemic period of allograft rejection, and infection showed a special predilection for the poorly perfused anastomotic site. 

## Discussion

Among lung transplant patients, more than one in three deaths in the first year will be from non-cytomegalovirus (CMV)-related infections; yet few studies have examined the underlying risk factors for their development [15,23]. Among these non-CMV infections, *A. fumigatus* is a common cause of morbidity and a potent predictor for increased mortality [5,6]. Putative risk factors include the degree of immune suppression, the route of pathogen exposure, the degree of airway ciliary dysfunction, and the absence of an effective cough [15]. Our current studies show for the first time that microvascular ischemia also may contribute to an increased risk of *A. fumigatus* invasion. 

The proclivity for *Aspergillus* survival at ischemic sites has been suggested by previous studies and by the well-described angiotropic nature of the fungus. Grahl and colleagues have shown, in murine models of IPA, that *Aspergillus* growth occurs in hypoxic conditions [11]. Using a hypoxia-detecting agent, pimonidazole hydrochloride, they demonstrated that invasive *Aspergillus* encounters severe hypoxia <10 mmHg in the lung and thrives in this microenvironment [11,24]. In our study a similar level of hypoxia was encountered only in infected animals at day 12 post-transplant (7.1 mmHg). The ability of *Aspergillus* to survive and thrive in hypoxic tissues provides a biological mechanism by which the mould may adapt to the varying ischemic conditions present in the current OTT model. In spite of this growing understanding, the host-pathogen interaction at the microvascular level is relatively unknown.

In our current study, *A. fumigatus* infection localized to the donor-recipient anastomosis regardless of the route of infection, level of ischemia or the presence of an alloimmune response. In 83% (25/30) of all animals infected, hyphal elements were seen primarily at the junction between the donor and recipient trachea. Thus, the phenotype of our murine model replicates the anatomic diathesis for the anastomotic infections observed clinically in lung transplant recipients. Several possible explanations may account for this finding including: the anatomic narrowing at this site, the tissue redundancy occurring as a consequence of the surgical ‘telescoping’ of one airway end tucked into the other end, the presence of foreign suture material and/or the attraction of the mould to an unidentified factor, present preferentially at the anastomosis. To evaluate one of these alternative hypotheses for anastomotic infection, in our preliminary experiments, we placed additional suture material in the middle of the allograft and on the recipient trachea above and below the cephalad and caudad anastomosis. We also placed suture material in non-transplanted controls. In none of these animals did infection appear to localize to the extra suture material within the graft or in the recipient trachea (data not shown). Thus, we suspect that suture material in the graft does not explain the localization of the infection at the anastomoses. 

Our research group has shown that the OTT anastomosis is a potent area of angiogenesis dependent on HIF-1α and *Sdf1*, a chemokine involved in the recruitment of angiogenic cells to hypoxic areas [17]. Through gene transfer experiments we have shown that HIF-1α overexpression by the allograft promotes microvascular stabilization, prolonging allograft perfusion to day 12 post-transplantation and accelerates neovascularization of the graft [17]. The predilection for an *A. fumigatus* anastomotic infection, in conjunction with our primary observation of progressive invasion with increasing ischemia, similarly suggests the presence of a factor that recruits *Aspergillus* to areas of ischemia and hypoxia. Teleologically, such a host-pathogen interaction may be explained by the need of the pathogen to seek nutrient sources. In the case of the saprophytic mould, the nutrient source would be decaying organic tissue. Hence, as the available blood supply wanes in an ischemic graft, the fungal invasion of the tissues increases. Extending this line of reasoning, proangiogenic factors upregulated in severe hypoxia such as HIF-1α may modulate the invasive nature of the fungus. Although the role of HIF in fungal pathogenesis is not well studied, it plays a key role in the host’s innate immune response to bacterial microorganisms [24-26]. Along these lines, *Chlamydia* has been demonstrated to down-regulate HIF-1α in order to gain a virulence advantage [26]. Currently, our research group is exploring the possibility that HIF may be a potent modulator of *A. fumigatus* invasion.

Finally, we show that *in vivo Aspergillus* infection correlated with decreases in blood perfusion of the anastomosis compared with the midtrachea in allograft animals. Significant differences in blood perfusion in the midtrachea and the donor-recipient anastomosis were only observed in the allograft animals at day 5 and 8 using the Doppler flowmetry probe. For infected animals at day 12 and in non-infected allografts no differences were observed. Similarly, in infected and non-infected syngeneic transplants no differences were observed. A lack of perfusion differences in syngeneic animals may be explained by a lower rate of infection overall observed in syngeneic compared to allogeneic animals (75% and 91%, respectively), as well as a difference in invasive disease (17% and 60%, respectively). Alternatively, syngeneic animals may have a more robust vasculature due to a lack of alloimmune-mediated vascular rejection [18]. Immune-mediated rejection may make allogeneic transplants more susceptible to the antiangiogenic or prothrombotic effects of the fungus. When evaluated by FITC-lectin, no clear differences were discernible between infected and non-infected animals. The difference between the two types of perfusion assays may be accounted for by the fact that FITC-lectin represents only a small portion of the vessels that are perfused and is uniquely informative about vessel architecture. By distinction, laser Doppler flowmetry measures “red blood cell flux,” which equals the product of the concentration of red blood cells moving in a tissue volume (0.3 mm^3^ to 0.75 mm^3^) and the mean velocity of these cells (www.oxford-optronix.com/pdfs/Technical/LDFprinciples.pdf). Thus, laser Doppler flowmetry because it measures a rate of red blood cell flow in a volume of tissue equivalent to the diameter of the graft, represents a more global and dynamic perfusion measurement of the tracheal segment than does FITC-lectin. Grossly the FITC-lectin perfusion studies did demonstrate marked dilation of midtracheal vessels compared to the anastomosis a finding that was consistent with the laser Doppler measurements. 

It has been long recognized that *A. fumigatus* may modulate the host’s microvasculature through vascular invasion, thrombosis, and more recently through the production of antiangiogenic factors [12,27]. Invasion of the vascular endothelial lumen has been shown to increase the release of tissue factor [27]. Tissue factor-mediated activation of the clotting cascade has been implicated in the vascular thrombosis that is well described in IPA [27]. *Aspergillus*-mediated platelet activation may also contribute to microvascular ischemia. RØdland et al., have demonstrated that *Aspergillus* may induce the expression of membrane-bound (CD63, CD26P) and soluble (RANTES, CD40L, DKK-1) platelet activating factors, resulting in thrombosis [28]. Interestingly, DKK-1 also is a potent regulator of the Wnt signaling pathway that plays a role in the regulation of inflammation and angiogenesis [28]. Ben-Ami and colleagues have shown that *A. fumigatus* produces a variety of metabolites, including gliotoxin, that inhibit angiogenesis [12]. Although vascular occlusion is a well-known feature radiographically and clinically, at present little is known of the signaling pathways that drive the mould’s antiangiogenic behavior. 

In previous work, we demonstrated that microvascular ischemia may be a diathesis for chronic rejection in lung transplant recipients [13]. It is possible that bronchial artery revascularization at the time of transplantation could limit the development of chronic rejection, and, similarly, a positive role for the bronchial circulation in maintaining the host defense mechanism has been posited [13,15,18]. The clinical relevance of our study could extend beyond lung transplantation to other forms of *Aspergillus* lung disease. One important difference among the patients that develop disparate forms of *Aspergillus* lung diseases is the degree of airway ischemia related to an abrupted bronchial circulation. For cystic fibrosis and asthmatic patients, the bronchial arterial network is robust [15]. It is speculated that vascular endothelial growth factor (VEGF), a downstream mediator for HIF, may cause the hypervascularity of the airways in asthmatics [29]. As predicted by our model, in these patients *A. fumigatus* typically displays a non-invasive phenotype. For a patient with COPD, in addition to architectural destruction, there is a slowly progressive destruction of the microvasculature and down regulation of VEGF [25,29]; in these patients a more indolent invasive infection may occur, chronic necrotizing aspergillosis. For lung transplant patients, ischemia due to the failure to restore the bronchial circulation may result in tracheal anastomotic infections early, and later, with *Aspergillus* colonization and with progressive microvascular loss associated with chronic rejection, in the development of IPA. An increased understanding of the role of the bronchial circulation in maintaining host defenses may provide novel treatments such as bronchial artery restoration or promotion of microvascular health and thus may possibly ameliorate these infections.

Several limitations of the current study deserve mention. To date, the literature hypothesizes that the mould itself drives angioinvasion [25], whereas the results of our study suggest that, in fact, the mould is responding to its microenvironment. Although the current study cannot unequivocally delineate the differential impact of the host or the pathogen, the overall perfusion kinetics in the Doppler flowmetry and FITC-lectin studies of both infected and non-infected allografts were similar. If *A. fumigatus* were the driving force behind the microvascular ischemia, we would have anticipated that that the infected animals would have had lower perfusion at all time points compared to non-infected allografts. Researchers also have reported differential expression of genes associated with angiogenesis and gliotoxin effects based on the type of immune suppression used in the murine model (i.e., corticosteroid model versus a cyclophosphamide induced neutropenic model) [12]. In the current study, mice were immune suppressed with a single dose of triamcinolone on the day of inoculation. Thus, the results of the current study may not be generalizable to findings from other murine models that typically administer corticosteroids at multiple time points or to neutropenic models of IPA. Additionally, we anticipate that the single dose of corticosteroid on the day of infection was unlikely to dramatically impact the alloimmune reaction. If this were the case, we would have anticipated changes in the tissue oxygen kinetics, BPU or FITC-lectin studies, as we have seen in our research group’s current work using immune modulatory therapy [14].

In conclusion, the study demonstrates three main findings: 1.) *A. fumigatus* invasion increases with progressive airway ischemia of the allograft, 2.) anastomotic infection predominates regardless of the allogeneic or syngeneic status of the transplant, route of infection or degree of airway ischemia, and 3.) *A. fumigatus* infection correlated with regional perfusion differences in the allogeneic transplanted trachea. Future work is needed to delineate putative chemo-attractant signals associated with angiogenesis that may explain *A. fumigatus* tropism into ischemic areas. Additionally, the model may allow delineation of the relative contribution of antiangiogenic factors versus thrombotic factors and the signaling pathways involved in modulating the host’s microvascular health. Such studies may prevent the occurrence or improve the treatment of *Aspergillus*-related lung diseases. 

## Supporting Information

Figure S1 **Experimental approach: intra-tracheal injection.** Animals were inoculated with *A. fumigatus* (3-4 x 10^8^ conidia/ml in 40 μl volume). All animals were euthanized 2 days after the day of infection as denoted by the letter “X”. All animals received triamcinolone acetonide (40mg/kg) on day of infection.
(TIF)

Figure S2 **Semiquantitative scale histopathological definitions.** Degree of fungal invasion and fungal burden were measured based on a 4-point semiquantitative scale. (**A**) Degree of fungal invasion was semiquantitatively graded: 1 (minimal) invasion of the epithelial layer, 2 (mild) invasion of the subepithelial layer, 3 (moderate), invasion to the depth of the cartilaginous tracheal ring, and 4 (severe), invasion through the tracheal wall (20X- 40X magnification). (**B**) Degree of fungal burden was determined using a semiquantitative scoring system as follows: 0, no fungal elements, 1 (minimal), fungal hyphae in less than 25% of the luminal area, 2 (mild) hyphae occluding 25% to 49% of the tracheal area, 3 (moderate) hyphae 50% to 74% of tracheal luminal area, and 4 (severe) greater than 75% occlusion of the tracheal luminal area (10X magnification). “*C*” denotes cartilaginous ring; “*E*” specifies epithelial layer; “*EL*” denotes extra-luminal space; “*SE*” specifies subepithelial layer; “*TL*” denotes tracheal lumen.
(TIF)

Figure S3 **No clear evidence of increasing disseminated infection by day post-allotransplantation.** (**A**) CFU (log_10_ CFU per gram organ, n = 5 animals/time period) of lung and kidney samples over time. (**B**) qPCR studies (log_10_ conidial equivalents per gram organ, n = 5 animals/time period) of lung and kidney samples over time. Although there was an increase in local invasion by increasing day post-transplantation, we did not demonstrate a similar increase in disseminated disease.
(TIF)
