# Supplementary figures and images for: Aspergillus fumigatus Invasion Increases with Progressive Airway Ischemia
Source: PLoS One. 2013 Oct 14;8(10):e77136. doi: 10.1371/journal.pone.0077136 (PMC3796538; doi:10.1371/journal.pone.0077136)

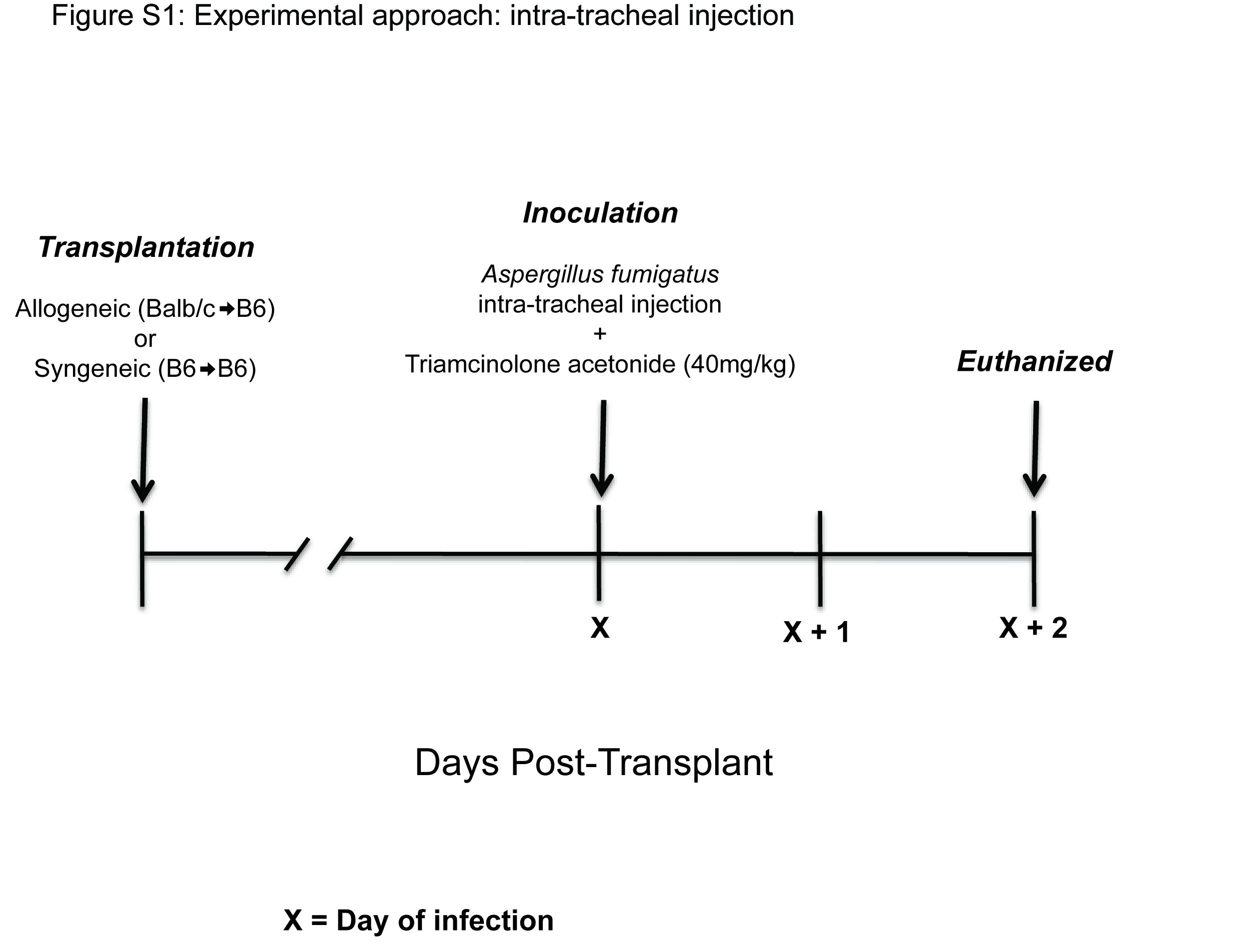

Supplement: Figure S1 — Experimental approach: intra-tracheal injection. Animals were inoculated with A. fumigatus (3-4 x 10^8^ conidia/ml in 40 μl volume). All animals were euthanized 2 days after the day of infection as denoted by the letter “X”. All animals received triamcinolone acetonide (40mg/kg) on day of infection. (TIF) [file pone.0077136.s001.tif]

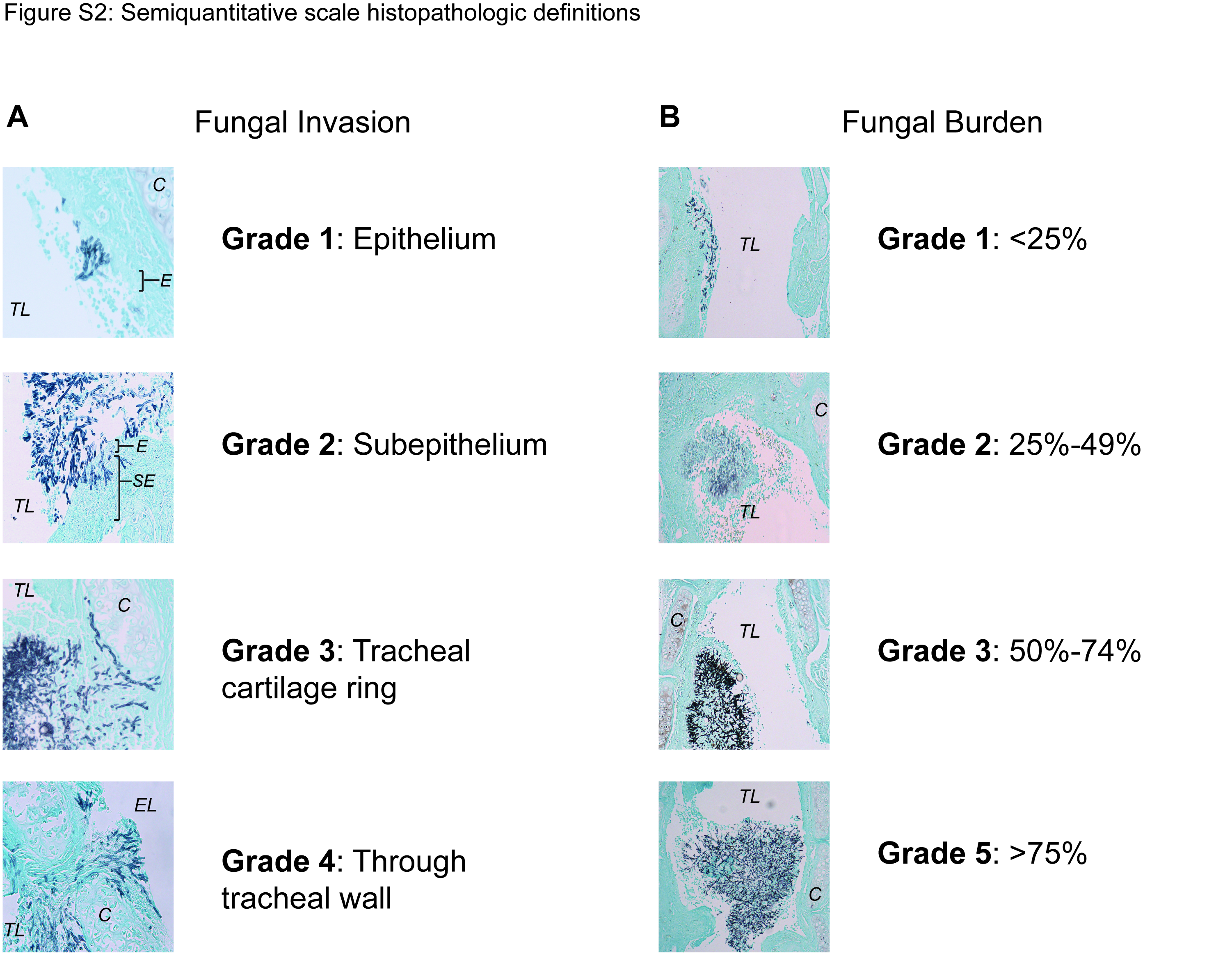

Supplement: Figure S2 — Semiquantitative scale histopathological definitions. Degree of fungal invasion and fungal burden were measured based on a 4-point semiquantitative scale. (A) Degree of fungal invasion was semiquantitatively graded: 1 (minimal) invasion of the epithelial layer, 2 (mild) invasion of the subepithelial layer, 3 (moderate), invasion to the depth of the cartilaginous tracheal ring, and 4 (severe), invasion through the tracheal wall (20X- 40X magnification). (B) Degree of fungal burden was determined using a semiquantitative scoring system as follows: 0, no fungal elements, 1 (minimal), fungal hyphae in less than 25% of the luminal area, 2 (mild) hyphae occluding 25% to 49% of the tracheal area, 3 (moderate) hyphae 50% to 74% of tracheal luminal area, and 4 (severe) greater than 75% occlusion of the tracheal luminal area (10X magnification). “C” denotes cartilaginous ring; “E” specifies epithelial layer; “EL” denotes extra-luminal space; “SE” specifies subepithelial layer; “TL” denotes tracheal lumen. (TIF) [file pone.0077136.s002.tif]

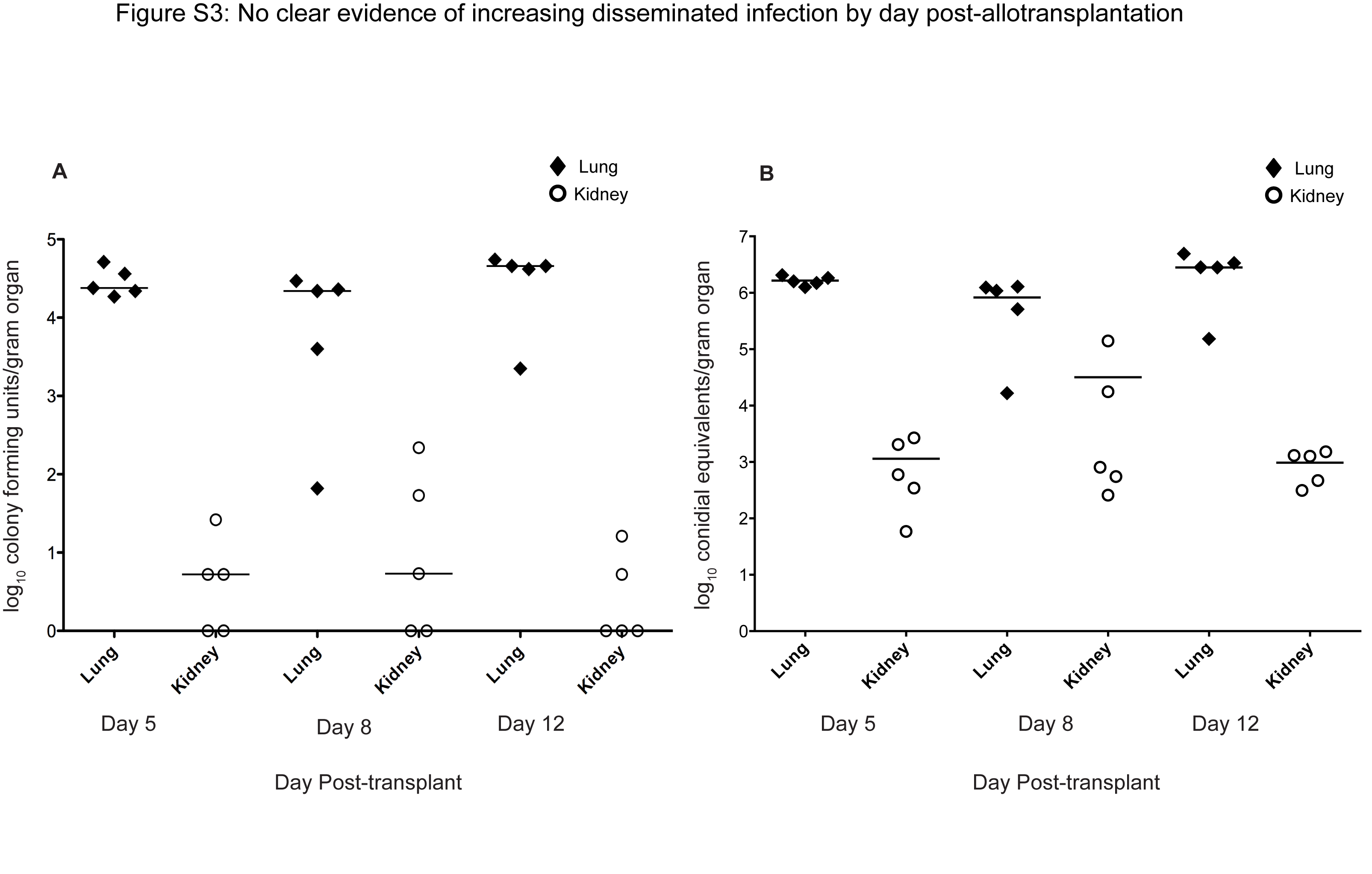

Supplement: Figure S3 — No clear evidence of increasing disseminated infection by day post-allotransplantation. (A) CFU (log10 CFU per gram organ, n = 5 animals/time period) of lung and kidney samples over time. (B) qPCR studies (log10 conidial equivalents per gram organ, n = 5 animals/time period) of lung and kidney samples over time. Although there was an increase in local invasion by increasing day post-transplantation, we did not demonstrate a similar increase in disseminated disease. (TIF) [file pone.0077136.s003.tif]
